# 3-(Adamantan-1-yl)-4-methyl-1-[(4-phenyl­piperazin-1-yl)meth­yl]-1*H*-1,2,4-triazole-5(4*H*)-thione dichloro­methane hemisolvate

**DOI:** 10.1107/S1600536812021393

**Published:** 2012-05-19

**Authors:** Ali A. El-Emam, Mohamed A. Al-Omar, Abdul-Malek S. Al-Tamimi, Seik Weng Ng, Edward R. T. Tiekink

**Affiliations:** a Department of Pharmaceutical Chemistry, College of Pharmacy, King Saud University, Riyadh 11451, Saudi Arabia; b Department of Chemistry, University of Malaya, 50603 Kuala Lumpur, Malaysia; c Chemistry Department, Faculty of Science, King Abdulaziz University, PO Box 80203 Jeddah, Saudi Arabia

## Abstract

The asymmetric unit of the title dichloro­methane hemisolvate, C_24_H_33_N_5_S·0.5CH_2_Cl_2_, comprises an adamantan­yl/triazole derivative and half a CH_2_Cl_2_ mol­ecule of crystallization; the latter is disordered about a twofold axis of symmetry. The piperazine ring has a chair conformation and the two N-bound substituents occupy equatorial positions. The piperazine residue is almost normal to the triazole ring [N—N—C—N torsion angle = −79.9 (3)°] so that to a first approximation, the mol­ecule has an L-shape. Linear supra­molecular chains parallel to [001] are formed *via* C—H⋯S inter­actions. Two such chains are linked into a double chain *via* C—H⋯Cl inter­actions involving the disordered CH_2_Cl_2_ mol­ecules of solvation.

## Related literature

For the diverse biological activities of adamantane derivatives, see: Al-Deeb *et al.* (2006 ▶); Al-Omar *et al.* (2010 ▶). For related adamantanyl structural studies, see: El-Emam *et al.* (2012*a* ▶,*b* ▶). For the preparation of one of the precursor mol­ecules, see: El-Emam & Ibrahim (1991 ▶).
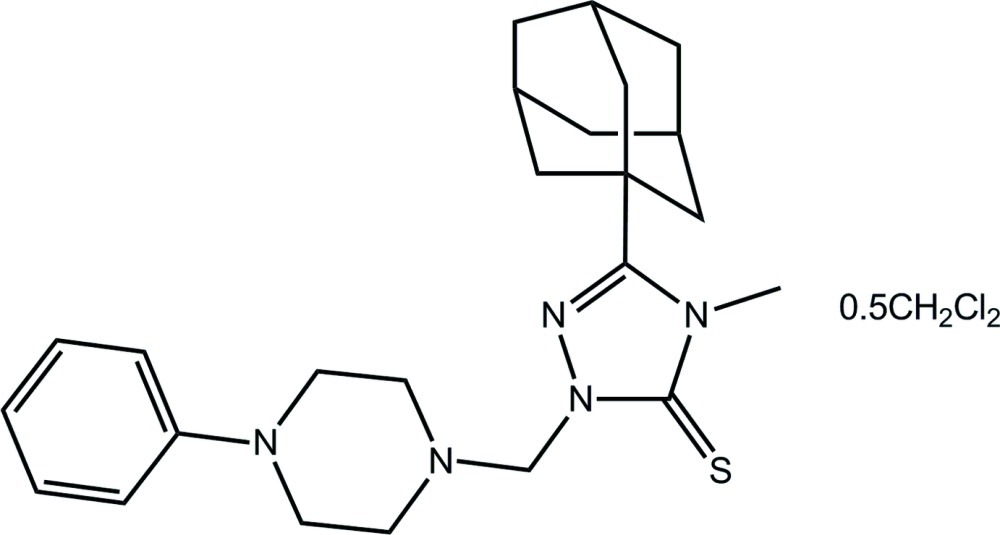


## Experimental

### 

#### Crystal data

2C_24_H_33_N_5_S·CH_2_Cl_2_
*M* *_r_* = 932.17
Orthorhombic, 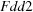

*a* = 66.8490 (16) Å
*b* = 22.1076 (4) Å
*c* = 6.5109 (1) Å
*V* = 9622.3 (3) Å^3^
*Z* = 8
Cu *K*α radiation
μ = 2.38 mm^−1^
*T* = 100 K
0.30 × 0.20 × 0.10 mm

#### Data collection

Agilent SuperNova Dual diffractometer with an Atlas detector
Absorption correction: multi-scan (*CrysAlis PRO*; Agilent, 2011 ▶) *T*_min_ = 0.752, *T*_max_ = 1.000
18884 measured reflections
4509 independent reflections
4437 reflections with *I* > 2σ(*I*)
*R*_int_ = 0.025

#### Refinement

*R*[*F* ^2^ > 2σ(*F* ^2^)] = 0.045
*wR*(*F* ^2^) = 0.126
*S* = 1.09
4509 reflections
299 parameters
19 restraints
H-atom parameters constrained
Δρ_max_ = 0.41 e Å^−3^
Δρ_min_ = −1.00 e Å^−3^
Absolute structure: Flack (1983 ▶), 1772 Friedel pairs
Flack parameter: −0.002 (17)

### 

Data collection: *CrysAlis PRO* (Agilent, 2011 ▶); cell refinement: *CrysAlis PRO*; data reduction: *CrysAlis PRO*; program(s) used to solve structure: *SHELXS97* (Sheldrick, 2008 ▶); program(s) used to refine structure: *SHELXL97* (Sheldrick, 2008 ▶); molecular graphics: *ORTEP-3* (Farrugia, 1997 ▶) and *DIAMOND* (Brandenburg, 2006 ▶); software used to prepare material for publication: *publCIF* (Westrip, 2010 ▶).

## Supplementary Material

Crystal structure: contains datablock(s) global, I. DOI: 10.1107/S1600536812021393/hg5226sup1.cif

Structure factors: contains datablock(s) I. DOI: 10.1107/S1600536812021393/hg5226Isup2.hkl

Supplementary material file. DOI: 10.1107/S1600536812021393/hg5226Isup3.cml

Additional supplementary materials:  crystallographic information; 3D view; checkCIF report

## Figures and Tables

**Table 1:** Hydrogen-bond geometry (Å, °)

*D*—H⋯*A*	*D*—H	H⋯*A*	*D*⋯*A*	*D*—H⋯*A*
C14—H14*A*⋯S1^i^	0.99	2.85	3.803 (3)	162
C16—H16*A*⋯Cl1	0.99	2.73	3.589 (4)	146

Symmetry code: (i) 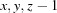.

**Fig. 1.**
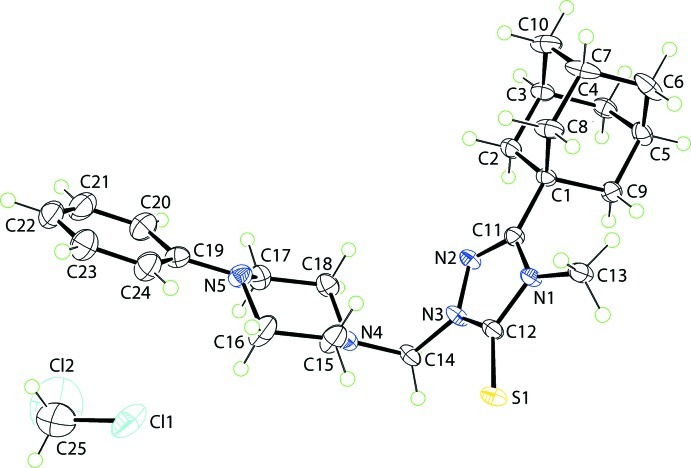
The molecular structures of the molecules comprising (I) showing the atom-labelling scheme and displacement ellipsoids at the 70% probability level. The CH2Cl2 molecule has 50% occupancy, being disordered over a twofold axis.

**Fig. 2.**
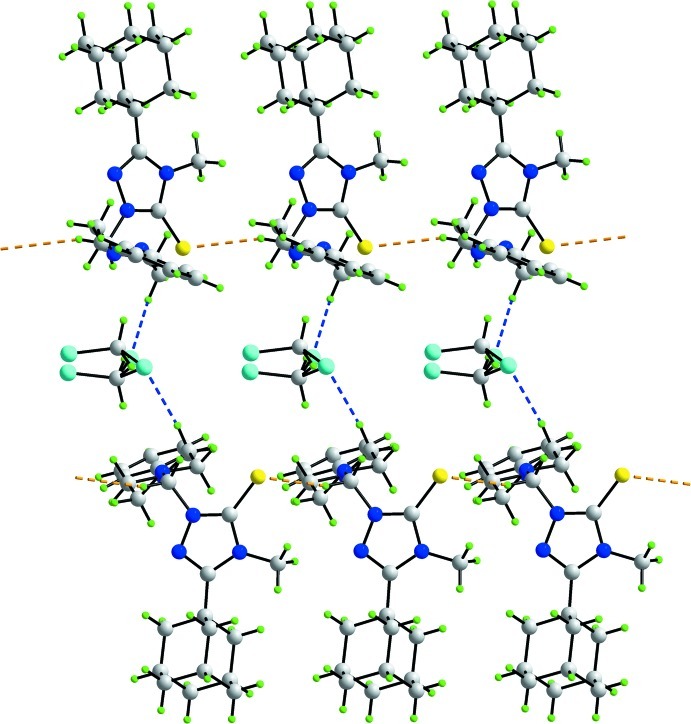
A view of the linear supramolecular double chain in (I). The C—H···S and C—H···Cl contacts are shown as orange and blue dashed lines, respectively. The CH2Cl2 molecule is disordered over two position; both orientations are displayed.

## supplementary crystallographic information

### Comment

In continuation of our interest in the chemical and pharmacological properties of adamantane derivatives, motivated by their putative biological activities (Al-Deeb *et al.*, 2006; Al-Omar *et al.*, 2010), and as part of on-going structural studies (El-Emam *et al.*, 2012*a*,*b*), we synthesized the title compound (I) as a potential chemotherapeutic agent. Herein, we describe the crystal and molecular structure of (I).

The asymmetric unit of (I), Fig. 1, comprises an adamantanyl/triazole derivative and half a CH_2_Cl_2_ molecule of crystallization. In the organic molecule, the piperazinyl ring has a chair conformation and the two N-bound substituents occupy equatorial positions. The piperazinyl residue is almost normal to the triazole ring with the N2—N3—C14—N4 torsion angle being -79.9 (3)°. To a first approximation, the molecule has an *L*-shape, as found recently in the 2-hydroxybenzylideneamino derivative (El-Emam *et al.*, 2012*a*).

In the crystal packing, linear supramolecular chains parallel to [001] are formed *via* C—H···S interactions, Table 1. These are linked into a double chain *via* C—H···Cl interactions involving the disordered CH_2_Cl_2_ molecules of solvation, Fig. 2 and Table 1.

### Experimental

A mixture of 3-(1-adamantyl)-4-methyl-4*H*-1,2,4-triazole-5-thiol (499 mg, 2 mmol), prepared according to the literature method (El-Emam & Ibrahim, 1991), 1-phenylpiperazine (325 mg, 2 mmol) and 37% formaldehyde solution (1 ml), in ethanol (8 ml), was heated under reflux for 15 min. when a clear solution was obtained. Stirring was continued for 12 h. at room temperature and the mixture was allowed to stand overnight. Cold water (5 ml) was slowly added and the mixture was stirred for 20 min. The precipitated crude product was filtered, washed with water, dried, and crystallized from ethanol to yield 635 mg (75%) of the title compound as colourless crystals. *M*.pt: 423–425 K. Single crystals suitable for X-ray analysis were obtained by slow evaporation of its CH_2_Cl_2_:EtOH solution held at room temperature (1:1; 5 ml). ^1^H NMR (CDCl_3_, 500.13 MHz): δ 1.77–1.84 (m, 6H, adamantane-H), 2.14 (s, 6H, adamantane-H), 2.27 (s, 3H, adamantane-H), 3.05 (s, 4H, piperazine-H), 3.27 (s, 4H, piperazine-H), 3.82 (s, 3H, CH_3_), 5.20 (s, 2H, CH_2_), 6.85–6.93 (m, 3H, Ar—H), 7.25–7.33 (m, 2H, Ar—H). ^13^C NMR (CDCl_3_, 125.76 MHz): δ 27.84, 33.97, 36.31, 39.02 (adamantane-C), 31.58 (CH_3_), 49.34, 50.40 (piperazine-C), 69.23 (CH_2_), 116.27, 119.86, 129.10, 151.33 (Ar—C), 156.31 (triazole C-5), 169.53 (C═S).

### Refinement

The H-atoms were placed in calculated positions [and C—H = 0.95 to 1.00 Å, *U*_iso_(H) = 1.2–1.5*U*_eq_(C)] and were included in the refinement in the riding model approximation. A number of reflections, *i.e*. (8 0 0), (18 2 0), (6 2 0), (10 2 0) and (2 2 0), were omitted from the final cycles of refinement owing to poor agreement. The maximum and minimum residual electron density peaks of 0.41 and 1.00 e Å^-3^, respectively, were located 0.62 Å and 0.63 Å from the Cl1 and Cl2 atoms, respectively.

### Crystal data

**Table:**

2C_24_H_33_N_5_S·CH_2_Cl_2_	*F*(000) = 3984
*M**_r_* = 932.17	*D*_x_ = 1.287 Mg m^−^^3^
Orthorhombic, *F**d**d*2	Cu *K*α radiation, λ = 1.54184 Å
Hall symbol: F 2 -2d	Cell parameters from 10015 reflections
*a* = 66.8490 (16) Å	θ = 2.6–76.4°
*b* = 22.1076 (4) Å	µ = 2.38 mm^−^^1^
*c* = 6.5109 (1) Å	*T* = 100 K
*V* = 9622.3 (3) Å^3^	Prism, colourless
*Z* = 8	0.30 × 0.20 × 0.10 mm

### Data collection

**Table:**

Agilent SuperNova Dual diffractometer with an Atlas detector	4509 independent reflections
Radiation source: SuperNova (Cu) X-ray Source	4437 reflections with *I* > 2σ(*I*)
Mirror monochromator	*R*_int_ = 0.025
Detector resolution: 10.4041 pixels mm^-1^	θ_max_ = 76.6°, θ_min_ = 2.6°
ω scan	*h* = −84→80
Absorption correction: multi-scan (*CrysAlis PRO*; Agilent, 2011)	*k* = −25→27
*T*_min_ = 0.752, *T*_max_ = 1.000	*l* = −7→8
18884 measured reflections	

### Refinement

**Table:**

Refinement on *F*^2^	Secondary atom site location: difference Fourier map
Least-squares matrix: full	Hydrogen site location: inferred from neighbouring sites
*R*[*F*^2^ > 2σ(*F*^2^)] = 0.045	H-atom parameters constrained
*wR*(*F*^2^) = 0.126	*w* = 1/[σ^2^(*F*_o_^2^) + (0.0825*P*)^2^ + 16.7617*P*] where *P* = (*F*_o_^2^ + 2*F*_c_^2^)/3
*S* = 1.09	(Δ/σ)_max_ = 0.002
4509 reflections	Δρ_max_ = 0.41 e Å^−^^3^
299 parameters	Δρ_min_ = −1.00 e Å^−^^3^
19 restraints	Absolute structure: Flack (1983), 1772 Friedel pairs
Primary atom site location: structure-invariant direct methods	Flack parameter: −0.002 (17)

### Special details

**Table:**

Geometry. All e.s.d.'s (except the e.s.d. in the dihedral angle between two l.s. planes) are estimated using the full covariance matrix. The cell e.s.d.'s are taken into account individually in the estimation of e.s.d.'s in distances, angles and torsion angles; correlations between e.s.d.'s in cell parameters are only used when they are defined by crystal symmetry. An approximate (isotropic) treatment of cell e.s.d.'s is used for estimating e.s.d.'s involving l.s. planes.
Refinement. Refinement of *F*^2^ against ALL reflections. The weighted *R*-factor *wR* and goodness of fit *S* are based on *F*^2^, conventional *R*-factors *R* are based on *F*, with *F* set to zero for negative *F*^2^. The threshold expression of *F*^2^ > σ(*F*^2^) is used only for calculating *R*-factors(gt) *etc*. and is not relevant to the choice of reflections for refinement. *R*-factors based on *F*^2^ are statistically about twice as large as those based on *F*, and *R*- factors based on ALL data will be even larger.

### Fractional atomic coordinates and isotropic or equivalent isotropic displacement parameters (Å^2^)

**Table:**

	*x*	*y*	*z*	*U*_iso_*/*U*_eq_	Occ. (<1)
S1	0.106131 (8)	0.22602 (2)	0.50061 (10)	0.02473 (15)	
N1	0.09696 (3)	0.34338 (8)	0.4049 (3)	0.0198 (4)	
N2	0.08323 (3)	0.33690 (8)	0.0966 (3)	0.0218 (4)	
N3	0.08877 (3)	0.28044 (8)	0.1693 (3)	0.0219 (4)	
N4	0.06533 (3)	0.20162 (8)	0.0489 (3)	0.0223 (4)	
N5	0.02334 (3)	0.19119 (9)	0.1251 (3)	0.0256 (4)	
C1	0.08658 (3)	0.44216 (9)	0.2235 (3)	0.0183 (4)	
C2	0.07497 (4)	0.45691 (10)	0.0251 (4)	0.0256 (5)	
H2A	0.0818	0.4383	−0.0939	0.031*	
H2B	0.0613	0.4396	0.0337	0.031*	
C3	0.07361 (4)	0.52562 (10)	−0.0064 (5)	0.0262 (5)	
H3	0.0661	0.5343	−0.1362	0.031*	
C4	0.09476 (4)	0.55174 (10)	−0.0222 (4)	0.0252 (5)	
H4A	0.0941	0.5960	−0.0450	0.030*	
H4B	0.1019	0.5333	−0.1400	0.030*	
C5	0.10617 (4)	0.53843 (11)	0.1778 (4)	0.0252 (5)	
H5	0.1199	0.5560	0.1685	0.030*	
C6	0.09524 (5)	0.56653 (11)	0.3591 (5)	0.0356 (6)	
H6A	0.0945	0.6110	0.3413	0.043*	
H6B	0.1026	0.5579	0.4875	0.043*	
C7	0.07398 (5)	0.54031 (11)	0.3735 (5)	0.0342 (6)	
H7	0.0668	0.5591	0.4924	0.041*	
C8	0.07510 (4)	0.47116 (10)	0.4044 (5)	0.0282 (5)	
H8A	0.0614	0.4541	0.4118	0.034*	
H8B	0.0820	0.4620	0.5352	0.034*	
C9	0.10775 (3)	0.46982 (10)	0.2081 (4)	0.0220 (5)	
H9A	0.1153	0.4610	0.3351	0.026*	
H9B	0.1150	0.4516	0.0909	0.026*	
C10	0.06261 (4)	0.55420 (11)	0.1747 (5)	0.0345 (6)	
H10A	0.0489	0.5377	0.1834	0.041*	
H10B	0.0617	0.5985	0.1550	0.041*	
C11	0.08839 (3)	0.37444 (9)	0.2426 (4)	0.0199 (4)	
C12	0.09709 (3)	0.28280 (10)	0.3552 (4)	0.0212 (4)	
C13	0.10506 (4)	0.36501 (10)	0.5994 (4)	0.0245 (5)	
H13A	0.1079	0.4084	0.5892	0.037*	
H13B	0.1175	0.3432	0.6308	0.037*	
H13C	0.0953	0.3580	0.7091	0.037*	
C14	0.08542 (3)	0.22529 (9)	0.0446 (4)	0.0227 (5)	
H14A	0.0890	0.2344	−0.0997	0.027*	
H14B	0.0947	0.1934	0.0935	0.027*	
C15	0.05794 (4)	0.18967 (13)	0.2540 (5)	0.0345 (6)	
H15A	0.0564	0.2283	0.3293	0.041*	
H15B	0.0677	0.1644	0.3292	0.041*	
C16	0.03797 (4)	0.15741 (15)	0.2457 (6)	0.0427 (8)	
H16A	0.0398	0.1168	0.1842	0.051*	
H16B	0.0328	0.1519	0.3870	0.051*	
C17	0.03096 (4)	0.20593 (11)	−0.0794 (4)	0.0278 (5)	
H17A	0.0212	0.2321	−0.1518	0.033*	
H17B	0.0326	0.1683	−0.1602	0.033*	
C18	0.05105 (4)	0.23845 (11)	−0.0635 (4)	0.0261 (5)	
H18A	0.0563	0.2468	−0.2030	0.031*	
H18B	0.0492	0.2776	0.0078	0.031*	
C19	0.00324 (4)	0.17111 (11)	0.1365 (4)	0.0281 (5)	
C20	−0.01038 (4)	0.17995 (14)	−0.0246 (5)	0.0366 (6)	
H20	−0.0061	0.1985	−0.1485	0.044*	
C21	−0.03030 (4)	0.16163 (14)	−0.0037 (6)	0.0426 (7)	
H21	−0.0393	0.1678	−0.1147	0.051*	
C22	−0.03716 (4)	0.13510 (14)	0.1726 (6)	0.0404 (7)	
H22	−0.0507	0.1227	0.1841	0.048*	
C23	−0.02392 (4)	0.12670 (14)	0.3341 (6)	0.0421 (7)	
H23	−0.0285	0.1087	0.4578	0.051*	
C24	−0.00399 (4)	0.14421 (14)	0.3179 (5)	0.0365 (6)	
H24	0.0048	0.1380	0.4304	0.044*	
Cl1	0.02109 (2)	0.01531 (9)	0.0431 (3)	0.0562 (4)	0.50
Cl2	0.0020 (2)	−0.0144 (4)	−0.3345 (6)	0.143 (3)	0.50
C25	0.00259 (15)	−0.0235 (4)	−0.0659 (17)	0.073 (2)	0.50
H25A	−0.0103	−0.0100	−0.0076	0.088*	0.50
H25B	0.0042	−0.0670	−0.0331	0.088*	0.50

### Atomic displacement parameters (Å^2^)

**Table:**

	*U*^11^	*U*^22^	*U*^33^	*U*^12^	*U*^13^	*U*^23^
S1	0.0326 (3)	0.0149 (2)	0.0267 (3)	0.0043 (2)	0.0009 (2)	0.0027 (2)
N1	0.0249 (9)	0.0129 (8)	0.0214 (10)	0.0011 (6)	−0.0011 (7)	−0.0014 (7)
N2	0.0278 (9)	0.0130 (8)	0.0248 (11)	−0.0014 (7)	0.0005 (8)	0.0021 (8)
N3	0.0324 (10)	0.0115 (8)	0.0218 (10)	−0.0020 (7)	0.0025 (8)	0.0002 (7)
N4	0.0244 (9)	0.0159 (8)	0.0266 (11)	−0.0011 (7)	0.0011 (8)	0.0007 (8)
N5	0.0237 (9)	0.0280 (9)	0.0252 (11)	0.0024 (8)	−0.0004 (8)	0.0046 (9)
C1	0.0202 (9)	0.0136 (9)	0.0212 (12)	0.0006 (7)	0.0027 (8)	0.0024 (8)
C2	0.0285 (11)	0.0162 (10)	0.0323 (14)	0.0006 (8)	−0.0061 (10)	0.0003 (10)
C3	0.0293 (11)	0.0166 (10)	0.0328 (14)	0.0045 (8)	−0.0026 (10)	0.0035 (10)
C4	0.0341 (12)	0.0160 (9)	0.0256 (13)	0.0007 (8)	0.0031 (10)	0.0045 (9)
C5	0.0311 (12)	0.0173 (10)	0.0274 (13)	−0.0061 (8)	−0.0017 (10)	0.0043 (9)
C6	0.0644 (18)	0.0135 (10)	0.0288 (15)	−0.0043 (10)	−0.0054 (13)	−0.0001 (10)
C7	0.0535 (16)	0.0183 (10)	0.0308 (15)	0.0140 (10)	0.0160 (12)	0.0015 (10)
C8	0.0339 (12)	0.0184 (10)	0.0323 (15)	0.0062 (9)	0.0139 (10)	0.0059 (10)
C9	0.0211 (10)	0.0189 (10)	0.0259 (14)	−0.0019 (7)	0.0023 (8)	0.0045 (9)
C10	0.0377 (13)	0.0223 (11)	0.0436 (16)	0.0123 (10)	0.0102 (12)	0.0069 (11)
C11	0.0197 (9)	0.0166 (9)	0.0234 (12)	−0.0002 (7)	0.0010 (8)	0.0011 (9)
C12	0.0250 (10)	0.0136 (9)	0.0252 (12)	−0.0006 (8)	0.0027 (9)	−0.0018 (9)
C13	0.0323 (12)	0.0175 (10)	0.0239 (12)	0.0049 (8)	−0.0056 (10)	−0.0023 (9)
C14	0.0293 (11)	0.0151 (9)	0.0239 (13)	−0.0028 (8)	0.0054 (9)	−0.0037 (9)
C15	0.0240 (11)	0.0448 (14)	0.0348 (15)	−0.0005 (10)	−0.0018 (11)	0.0222 (13)
C16	0.0234 (11)	0.0544 (17)	0.0504 (19)	−0.0021 (11)	−0.0016 (12)	0.0336 (16)
C17	0.0292 (12)	0.0297 (12)	0.0244 (12)	−0.0018 (9)	−0.0028 (9)	0.0004 (10)
C18	0.0327 (12)	0.0231 (10)	0.0224 (12)	−0.0025 (9)	−0.0018 (9)	0.0044 (9)
C19	0.0243 (11)	0.0265 (11)	0.0335 (15)	0.0049 (9)	0.0024 (10)	−0.0044 (10)
C20	0.0304 (12)	0.0439 (14)	0.0356 (17)	0.0019 (11)	−0.0042 (11)	0.0003 (13)
C21	0.0277 (12)	0.0500 (16)	0.0500 (19)	0.0048 (11)	−0.0068 (13)	−0.0122 (15)
C22	0.0241 (12)	0.0406 (15)	0.057 (2)	−0.0005 (10)	0.0028 (12)	−0.0154 (14)
C23	0.0312 (13)	0.0454 (16)	0.0498 (19)	−0.0028 (11)	0.0101 (13)	0.0008 (14)
C24	0.0279 (12)	0.0425 (15)	0.0391 (17)	−0.0006 (10)	0.0028 (11)	0.0028 (13)
Cl1	0.0308 (7)	0.0813 (11)	0.0563 (11)	0.0133 (7)	−0.0006 (6)	−0.0114 (9)
Cl2	0.150 (5)	0.182 (8)	0.096 (2)	−0.015 (6)	0.001 (3)	−0.007 (3)
C25	0.074 (5)	0.059 (4)	0.086 (6)	0.008 (4)	−0.001 (5)	−0.005 (4)

### Geometric parameters (Å, º)

**Table:**

S1—C12	1.684 (2)	C8—H8A	0.9900
N1—C12	1.378 (3)	C8—H8B	0.9900
N1—C11	1.384 (3)	C9—H9A	0.9900
N1—C13	1.458 (3)	C9—H9B	0.9900
N2—C11	1.308 (3)	C10—H10A	0.9900
N2—N3	1.386 (2)	C10—H10B	0.9900
N3—C12	1.333 (3)	C13—H13A	0.9800
N3—C14	1.482 (3)	C13—H13B	0.9800
N4—C14	1.442 (3)	C13—H13C	0.9800
N4—C15	1.448 (3)	C14—H14A	0.9900
N4—C18	1.453 (3)	C14—H14B	0.9900
N5—C19	1.417 (3)	C15—C16	1.515 (3)
N5—C16	1.460 (3)	C15—H15A	0.9900
N5—C17	1.462 (3)	C15—H15B	0.9900
C1—C11	1.507 (3)	C16—H16A	0.9900
C1—C2	1.542 (3)	C16—H16B	0.9900
C1—C9	1.545 (3)	C17—C18	1.526 (3)
C1—C8	1.545 (3)	C17—H17A	0.9900
C2—C3	1.536 (3)	C17—H17B	0.9900
C2—H2A	0.9900	C18—H18A	0.9900
C2—H2B	0.9900	C18—H18B	0.9900
C3—C10	1.527 (4)	C19—C20	1.403 (4)
C3—C4	1.531 (3)	C19—C24	1.408 (4)
C3—H3	1.0000	C20—C21	1.398 (4)
C4—C5	1.538 (4)	C20—H20	0.9500
C4—H4A	0.9900	C21—C22	1.368 (5)
C4—H4B	0.9900	C21—H21	0.9500
C5—C6	1.521 (4)	C22—C23	1.386 (5)
C5—C9	1.533 (3)	C22—H22	0.9500
C5—H5	1.0000	C23—C24	1.392 (4)
C6—C7	1.538 (4)	C23—H23	0.9500
C6—H6A	0.9900	C24—H24	0.9500
C6—H6B	0.9900	Cl1—C25	1.664 (10)
C7—C10	1.532 (4)	Cl2—C25	1.761 (12)
C7—C8	1.544 (3)	C25—H25A	0.9900
C7—H7	1.0000	C25—H25B	0.9900
			
C12—N1—C11	107.82 (19)	C3—C10—H10B	109.8
C12—N1—C13	121.3 (2)	C7—C10—H10B	109.8
C11—N1—C13	130.84 (18)	H10A—C10—H10B	108.3
C11—N2—N3	104.64 (19)	N2—C11—N1	110.43 (18)
C12—N3—N2	112.72 (18)	N2—C11—C1	123.3 (2)
C12—N3—C14	126.35 (19)	N1—C11—C1	126.1 (2)
N2—N3—C14	120.92 (19)	N3—C12—N1	104.39 (19)
C14—N4—C15	113.7 (2)	N3—C12—S1	129.11 (17)
C14—N4—C18	113.51 (18)	N1—C12—S1	126.51 (19)
C15—N4—C18	110.06 (19)	N1—C13—H13A	109.5
C19—N5—C16	116.5 (2)	N1—C13—H13B	109.5
C19—N5—C17	116.6 (2)	H13A—C13—H13B	109.5
C16—N5—C17	111.7 (2)	N1—C13—H13C	109.5
C11—C1—C2	108.64 (18)	H13A—C13—H13C	109.5
C11—C1—C9	108.97 (17)	H13B—C13—H13C	109.5
C2—C1—C9	108.83 (19)	N4—C14—N3	115.42 (19)
C11—C1—C8	112.89 (18)	N4—C14—H14A	108.4
C2—C1—C8	107.53 (19)	N3—C14—H14A	108.4
C9—C1—C8	109.89 (19)	N4—C14—H14B	108.4
C3—C2—C1	110.5 (2)	N3—C14—H14B	108.4
C3—C2—H2A	109.5	H14A—C14—H14B	107.5
C1—C2—H2A	109.5	N4—C15—C16	110.7 (2)
C3—C2—H2B	109.5	N4—C15—H15A	109.5
C1—C2—H2B	109.5	C16—C15—H15A	109.5
H2A—C2—H2B	108.1	N4—C15—H15B	109.5
C10—C3—C4	109.9 (2)	C16—C15—H15B	109.5
C10—C3—C2	109.5 (2)	H15A—C15—H15B	108.1
C4—C3—C2	109.13 (18)	N5—C16—C15	111.7 (2)
C10—C3—H3	109.4	N5—C16—H16A	109.3
C4—C3—H3	109.4	C15—C16—H16A	109.3
C2—C3—H3	109.4	N5—C16—H16B	109.3
C3—C4—C5	109.2 (2)	C15—C16—H16B	109.3
C3—C4—H4A	109.8	H16A—C16—H16B	107.9
C5—C4—H4A	109.8	N5—C17—C18	110.5 (2)
C3—C4—H4B	109.8	N5—C17—H17A	109.6
C5—C4—H4B	109.8	C18—C17—H17A	109.6
H4A—C4—H4B	108.3	N5—C17—H17B	109.6
C6—C5—C9	109.7 (2)	C18—C17—H17B	109.6
C6—C5—C4	109.9 (2)	H17A—C17—H17B	108.1
C9—C5—C4	109.4 (2)	N4—C18—C17	110.37 (19)
C6—C5—H5	109.3	N4—C18—H18A	109.6
C9—C5—H5	109.3	C17—C18—H18A	109.6
C4—C5—H5	109.3	N4—C18—H18B	109.6
C5—C6—C7	109.7 (2)	C17—C18—H18B	109.6
C5—C6—H6A	109.7	H18A—C18—H18B	108.1
C7—C6—H6A	109.7	C20—C19—C24	117.6 (2)
C5—C6—H6B	109.7	C20—C19—N5	122.2 (3)
C7—C6—H6B	109.7	C24—C19—N5	120.1 (2)
H6A—C6—H6B	108.2	C21—C20—C19	120.3 (3)
C10—C7—C6	109.4 (2)	C21—C20—H20	119.8
C10—C7—C8	109.4 (2)	C19—C20—H20	119.8
C6—C7—C8	109.7 (2)	C22—C21—C20	121.7 (3)
C10—C7—H7	109.5	C22—C21—H21	119.2
C6—C7—H7	109.5	C20—C21—H21	119.2
C8—C7—H7	109.5	C21—C22—C23	118.7 (3)
C7—C8—C1	109.6 (2)	C21—C22—H22	120.7
C7—C8—H8A	109.8	C23—C22—H22	120.7
C1—C8—H8A	109.8	C22—C23—C24	121.1 (3)
C7—C8—H8B	109.8	C22—C23—H23	119.5
C1—C8—H8B	109.8	C24—C23—H23	119.5
H8A—C8—H8B	108.2	C23—C24—C19	120.6 (3)
C5—C9—C1	109.71 (18)	C23—C24—H24	119.7
C5—C9—H9A	109.7	C19—C24—H24	119.7
C1—C9—H9A	109.7	Cl1—C25—Cl2	112.3 (8)
C5—C9—H9B	109.7	Cl1—C25—H25A	109.1
C1—C9—H9B	109.7	Cl2—C25—H25A	109.1
H9A—C9—H9B	108.2	Cl1—C25—H25B	109.1
C3—C10—C7	109.3 (2)	Cl2—C25—H25B	109.1
C3—C10—H10A	109.8	H25A—C25—H25B	107.9
C7—C10—H10A	109.8		
			
C11—N2—N3—C12	0.2 (2)	C2—C1—C11—N1	−176.7 (2)
C11—N2—N3—C14	−179.6 (2)	C9—C1—C11—N1	64.9 (3)
C11—C1—C2—C3	−177.29 (19)	C8—C1—C11—N1	−57.5 (3)
C9—C1—C2—C3	−58.8 (2)	N2—N3—C12—N1	−0.1 (2)
C8—C1—C2—C3	60.2 (2)	N2—N3—C12—S1	−179.48 (17)
C1—C2—C3—C10	−60.5 (3)	C14—N3—C12—S1	0.3 (3)
C1—C2—C3—C4	59.9 (3)	C11—N1—C12—N3	0.0 (2)
C10—C3—C4—C5	59.7 (2)	C13—N1—C12—N3	−179.56 (19)
C2—C3—C4—C5	−60.5 (3)	C11—N1—C12—S1	179.40 (17)
C3—C4—C5—C6	−59.3 (2)	C13—N1—C12—S1	−0.2 (3)
C3—C4—C5—C9	61.3 (2)	C15—N4—C14—N3	−54.9 (3)
C9—C5—C6—C7	−60.9 (3)	C18—N4—C14—N3	71.9 (3)
C4—C5—C6—C7	59.5 (3)	C12—N3—C14—N4	100.3 (3)
C5—C6—C7—C10	−59.8 (3)	N2—N3—C14—N4	−79.9 (3)
C5—C6—C7—C8	60.2 (3)	C14—N4—C15—C16	−172.6 (2)
C10—C7—C8—C1	61.3 (3)	C18—N4—C15—C16	58.8 (3)
C6—C7—C8—C1	−58.6 (3)	C19—N5—C16—C15	−169.1 (3)
C11—C1—C8—C7	179.8 (2)	C17—N5—C16—C15	53.3 (3)
C2—C1—C8—C7	−60.4 (3)	N4—C15—C16—N5	−55.6 (3)
C9—C1—C8—C7	57.9 (3)	C19—N5—C17—C18	168.7 (2)
C6—C5—C9—C1	60.0 (3)	C16—N5—C17—C18	−53.8 (3)
C4—C5—C9—C1	−60.6 (3)	C14—N4—C18—C17	171.5 (2)
C11—C1—C9—C5	177.2 (2)	C15—N4—C18—C17	−59.8 (3)
C2—C1—C9—C5	58.9 (2)	N5—C17—C18—N4	57.1 (3)
C8—C1—C9—C5	−58.6 (3)	C16—N5—C19—C20	−152.3 (3)
C4—C3—C10—C7	−60.4 (3)	C17—N5—C19—C20	−16.8 (3)
C2—C3—C10—C7	59.5 (3)	C16—N5—C19—C24	31.2 (4)
C6—C7—C10—C3	59.9 (3)	C17—N5—C19—C24	166.7 (2)
C8—C7—C10—C3	−60.2 (3)	C24—C19—C20—C21	−0.9 (4)
N3—N2—C11—N1	−0.1 (2)	N5—C19—C20—C21	−177.4 (2)
N3—N2—C11—C1	175.2 (2)	C19—C20—C21—C22	0.4 (5)
C12—N1—C11—N2	0.1 (3)	C20—C21—C22—C23	0.4 (5)
C12—N1—C11—C1	−175.1 (2)	C21—C22—C23—C24	−0.6 (5)
C13—N1—C11—C1	4.4 (4)	C22—C23—C24—C19	0.1 (5)
C2—C1—C11—N2	8.7 (3)	C20—C19—C24—C23	0.6 (4)
C9—C1—C11—N2	−109.8 (2)	N5—C19—C24—C23	177.2 (3)
C8—C1—C11—N2	127.9 (2)		

### Hydrogen-bond geometry (Å, º)

**Table:**

*D*—H···*A*	*D*—H	H···*A*	*D*···*A*	*D*—H···*A*
C14—H14*A*···S1^i^	0.99	2.85	3.803 (3)	162
C16—H16*A*···Cl1	0.99	2.73	3.589 (4)	146

Symmetry code: (i) *x*, *y*, *z*−1.
